# GOToolBox: functional analysis of gene datasets based on Gene Ontology

**DOI:** 10.1186/gb-2004-5-12-r101

**Published:** 2004-11-26

**Authors:** David Martin, Christine Brun, Elisabeth Remy, Pierre Mouren, Denis Thieffry, Bernard Jacq

**Affiliations:** 1 Laboratoire de Génétique et Physiologie du Développement, IBDM, CNRS/INSERM/Université de la Méditerranée, Parc Scientifique de Luminy, case 907, 13288 Marseille, France; 2 Institut de Mathématiques de Luminy, Parc Scientifique de Luminy, 13288 Marseille, France

## Abstract

We have developed methods and tools based on the Gene Ontology (GO) resource allowing the identification of statistically over- or under-represented terms in a gene dataset; the clustering of functionally related genes within a set; and the retrieval of genes sharing annotations with a query gene. GO annotations can also be constrained to a slim hierarchy or a given level of the ontology. The source codes are available upon request, and distributed under the GPL license.

## Abstract 2

Tools are presented to identify Gene Ontology terms that are over- or under-represented in a dataset, to cluster genes by function and to find genes with similar annotations.

## Rationale

Since complete genome sequences have become available, the amount of annotated genes has increased dramatically. These advances have allowed the systematic comparison of the gene content of different organisms, leading to the conclusion that organisms share the majority of their genes with only relatively few species-specific genes. On this basis, one can develop strategies to infer gene annotations from model species to less experimentally tractable organisms. However, such functional inferences require the definition of species-independent annotation policies.

In this regard, the Gene Ontology consortium [1] has been created to develop a unified view of gene functional annotations for different model organisms. Three structured vocabularies (or ontologies) have been proposed, which allow the description of molecular functions, biological processes and cellular locations of any gene product, respectively. Whereas the majority of GO terms are common to several organisms, some of them are specific to a few organisms only, enabling the description of some aspects of gene function which are specific to few lineages only. Within each of these ontologies, the terms are organized in a hierarchical way, according to parent-child relationships in a directed acyclic graph (DAG). This allows a progressive functional description, matching the current level of experimental characterization of the corresponding gene product. The hierarchical organization of the gene ontology is particularly well adapted to computational processing and is used for the functional annotations of gene products of several model organisms such as budding yeast [2], *Drosophila *[3], mouse [4], nematode [5] and *Arabidopsis *[6]. More recently, GO annotations for human genes have been proposed in the context of the GOA project [7].

In parallel, the recent development of new high-throughput methods has generated an enormous amount of functional data and has motivated the development of dedicated analysis tools. For instance, one might wonder whether genes detected as being coexpressed in a DNA chip experiment are related in terms of molecular or cellular function. In practical terms, we address here the following generic questions. First, are there statistically over- or under-represented GO terms associated with a given gene set, compared to the distribution of these terms among the annotations of the complete genome? Second, among a particular gene set, are there closely functionally related gene subsets? And third, are there genes having GO similarities with a given probe gene?

**Figure 1:**
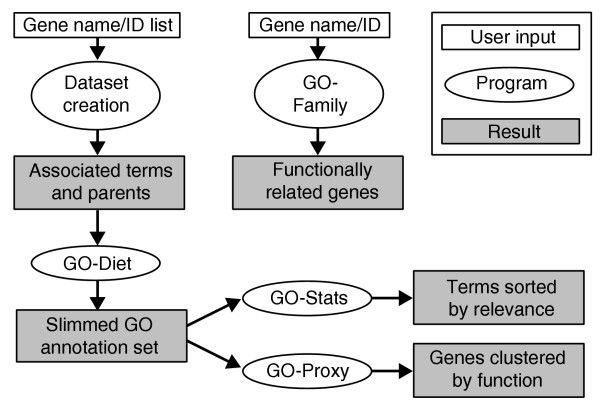
Flowchart of the GOToolBox programs.

To formulate such questions properly in a well defined mathematical framework, we have developed a set of methods and tools, collectively called GOToolBox, to process the GO annotations for any model organism for which they are available (Figure 1).

All the programs are written in PERL and use the CGI and DBI modules. All the ontology data and the gene-GO terms associations are taken from the GO consortium website. These data are structured in a PostGreSQL relational database, which is updated monthly. Statistics are calculated using the R statistical programming environment. The web implementation of the GOToolBox is accessible at [8].

## Features

In this section, we describe the five main functionalities of the GOToolBox suite. Two of them (GO-Proxy and GO-Family) are not encompassed by any other GO-processing tool currently available (see also 'Comparison of the GOToolBox with other GO-based analysis programs').

### Dataset creation

The first step in analyzing gene datasets consists in retrieving, for each individual gene of the dataset, all the corresponding GO terms and their parent terms using the Dataset creation program. The genomic frequency of each GO term associated with genes present in the dataset is then calculated. The resulting information is structured and stored in a data file, available for download on the GOToolBox server for one week. This file contains also the counts of terms within a reference gene dataset (genome or user-defined), and can then be used as an input for the GO-Stats and GO-Proxy programs described below.

### Ontology options

An optional tool, GO-Diet, allows either the reduction of the term dataset to a slim GO hierarchy (either one proposed by the GO consortium or a user-defined one) or the restriction of the considered terms to a chosen depth of the ontology. It is also possible to filter terms based on the way these have been assigned to the gene products (evidence code). This tool is useful to decrease the number of GO terms associated with a gene dataset, thereby facilitating the analysis of the results of programs described below, particularly when the input gene list and/or the number of associated GO terms is large. Note that the GO-Diet program can generate a gene-term association file in the TLF format, allowing the use of GO terms as gene labels with the TreeDyn tree drawing program [9]. The GO-Diet options are proposed in the Dataset-Creation form.

### GO term statistics

**Figure 2:**
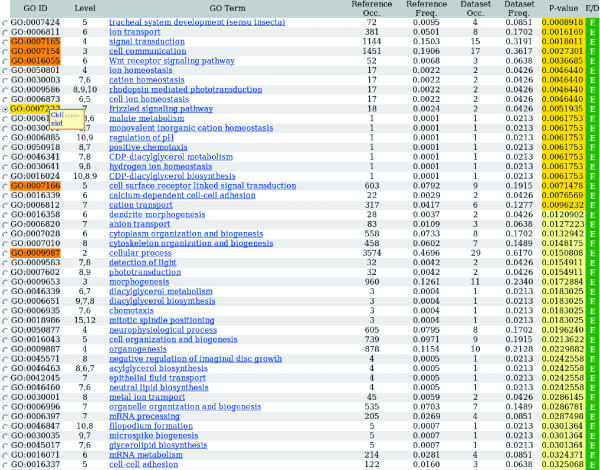
Typical output from the GO-Stats program. From the input of a group of *Drosophila *genes, GO-stat returns a series of GO terms associated with them (columns 1 and 3). The terms are ranked according to a *P*-value representing their statistical relevance (column 8). The output also lists additional useful information: column 2 describes the depth at which a given GO term is found in the GO hierarchy (note that some terms can be found at several levels simultaneously; for example, GO:0009586). Columns 4 and 6 list the numbers of genes annotated for a given term in the reference and the user sets, respectively. Columns 5 and 7 list the corresponding occurrence frequencies. Finally, the last column indicates whether a given GO term is enriched (E) or depleted (D), based on the term frequency ratio (column 7/column 5). Note that hyperlinks to GO terms definitions by the GO consortium are provided (underlined in column 3). In such an output, all GO terms associated with the input genes are listed in the table. To visualize the hierarchy between these terms, an interactive functional feature is provided with GO-Stats: by clicking on a term (radio button on the left of GO terms list), all its parent terms in the list are highlighted. Finally, when working in the program, moving the mouse pointer on the GO ID column will make all the genes associated with a given GO term appear in a box.

Frequencies of terms within the dataset are calculated and compared with reference frequencies (for example with genomic frequencies or with the frequencies of these terms in the complete list of genes spotted on an array). This procedure allows the delineation of enrichments or depletions of specific terms in the dataset. The probability of obtaining by chance a number *k *of annotated genes for a given term among a dataset of size *n*, knowing that the reference dataset contains *m *such annotated genes out of *N *genes, is then calculated. This test follows the hypergeometric distribution described in Equation 1:



where the random variable *X *represents the number of genes within a given gene subset, annotated with a given GO term. Implemented in the GO-Stats tool, this formula permits the automatic ranking of all annotation terms, as well as the evaluation of the significance of their occurrences within the dataset. An illustration of such an approach is given in 'Mining biological data'. A typical GO-Stats output is presented in Figure 2.

### GO-based gene clustering

The goal of the GO-Proxy tool is to group together functionally related genes on the basis of their GO terms. The rationale sustaining our method is that the more genes have common GO terms, and the less they have specific associated terms, the more likely they are to be functionally related. For any two genes of the gene set, the program calculates an annotation-based distance between genes, taking into account all GO terms that are common to the pair and terms which are specific to each gene. Indeed, any two genes can have 0, 1 or several shared GO terms (common terms) and a variable number of terms specific for each gene (specific terms). This distance is based on the Czekanowski-Dice formula (Equation 2):



In this formula, *x *and *y *denote two genes, *Terms(x) *and *Terms(y) *are the lists of their associated GO terms, *# *stands for 'number of ' and Δ for the symmetrical difference between the two sets. This distance formula emphasizes the importance of the shared GO terms by giving more weight to similarities than to differences. Consequently, for two genes that do not share any GO terms, the distance value is 1, the highest possible value, whereas for two genes sharing exactly the same set of GO terms, the distance value is 0, the lowest possible value. All possible binary pairs of genes from the dataset are considered, resulting in a distance matrix.

Next this matrix is processed with a clustering algorithm, such as the WPGMA algorithm, and a functional classification tree is drawn, in which the leaves correspond to input genes. On the basis of this tree, classes can be defined, for instance by using partition rules, and the statistical relevance of the terms associated with each class is calculated using the method described for GO-Stats. The Czekanowski-Dice distance and the corresponding clustering have already proved their effectiveness in delineating protein functional classes derived from the analysis of protein-protein interaction graphs [10].

### Finding GO-related genes

A last tool, GO-Family, aims at finding genes having shared GO terms with a user-entered gene, on the basis of a functional similarity calculation. It searches the genomes either of one or several supported species (five at the moment). Given an input gene name, the program retrieves the associated GO terms and compares them with those of all other genes by calculating a functional similarity percentage. The program then returns the list of similar genes, sorted by score. By similar genes, we mean either genes having more than one common associated term, or genes which have different associated terms but one or more common parent terms.

When measuring the similarity percentage *S *between the input gene A and another gene G, one can identify terms that are common to the two genes (Tc), and terms that are specific to A (Ta) and G (Tg). Three different similarity measures have been implemented and proposed to the user:

Si = (Tc/(Ta+Tc)) × 100     (3)

Sp = (Tc/(Ta+Tg+Tc)) × 100     (4)

**Figure 3:**
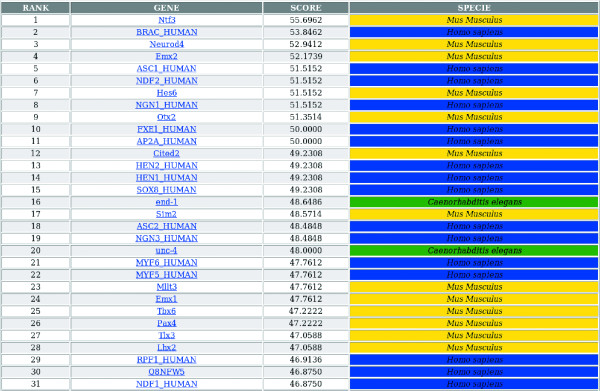
Typical output from the GO-Family program. In this figure, we have asked for all the genes from human, mouse and nematode that share more than 45% functional similarity with an input gene: the *Drosophila *gene *engrailed*. The output is composed of four columns: rank, name of similar gene, percentage of similarity and species from which the similar gene is issued.

Scd = (1 - ((Ta+Tg)/(Ta+Tg+2Tc))) × 100     (5)

respectively called similarity percentage relative to the input gene (*Si*), similarity percentage relative to the pair of genes (*Sp*) and Czekanowski-Dice proximity percentage (*Scd*). The results are then ranked by decreasing similarity values. A typical GO-Family output is presented in Figure 3.

## Mining biological data with the GOToolBox

In this section, we provide two examples showing how combinations of several GO analysis tools can be used to validate or further delineate gene functional classifications.

### Application of GOToolBox to the study of protein-protein interaction networks

**Figure 4:**
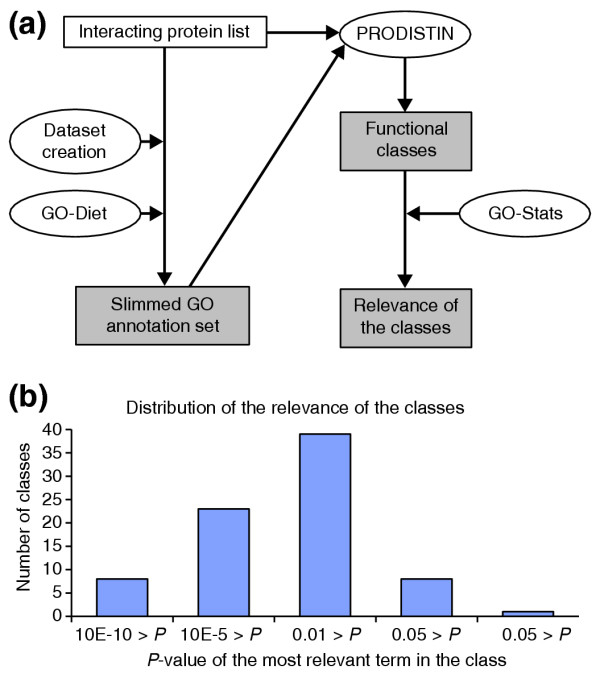
Use of the GOToolBox programs in the PRODISTIN framework. **(a) **Flowchart of the programs used in the PRODISTIN pipeline. The 'Dataset creation' program and GO-Diet are used to generate a slimmed protein annotation file in a suitable format (tlf). This tlf file can be used as input both for PRODISTIN and for the tree-visualization program TreeDyn (not shown in the figure). In a second step, when functional classes have been generated by PRODISTIN, the GO-Stats tool allows the evaluation of the relevance of the class annotation term. **(b) **Histograms showing the distribution of the relevance values for the 79 classes issued from PRODISTIN (probability is described in the Features section).

PRODISTIN [10] is a functional classification method for proteins, based on the analysis of a protein-protein interaction network, that aims to compare and predict a cellular role for proteins of unknown function. Given a set of proteins and a list of interactions between them, a distance is calculated between all possible pairs of proteins. A distance matrix is then generated, to which the NJ clustering algorithm is applied. A classification tree is then built, within which functional classes are defined, based on the annotation terms associated with the proteins involved in known biological processes. GO-Diet and GO-Stats are useful at two steps of the analysis (Figure 4a).

The first is to generate the GO annotation set necessary to define the functional classes of proteins. In this particular study devoted to the yeast interactome, the term dataset was fitted to the fourth ontology level using GO-Diet. We chose to work at this particular level because it was previously shown to provide a good representation of the complexity of the cellular functions of the proteins described by the biological process annotations [10]. The second step is to estimate the relevance of the annotations associated with the resulting classes using associated GO terms. The GO-Stats program can be used in this framework, using as reference dataset the list of proteins given as an input to PRODISTIN (Figure 4b).

As shown in Table 1, the classes issued from PRODISTIN can be associated to one or to several GO terms. In the latter case, the calculated annotation biases emphasize the most relevant terms for the functional assignment of the class (first row in Table 1), allowing the ranking of the annotation terms. When the class is associated with a single GO term (second and third rows in table 1), GO-Stats estimates the probability of obtaining a class with the same size and functional coherence associated by chance with this GO term. For instance, in Table 1, the term 'RNA metabolism' is clearly over-represented in the second class, whereas this is certainly not true in the case of the 'cell cycle' class.

### Functional clustering of sets of transcriptional factor targets

GO can also be used to split gene sets into coherent functional subclasses on the basis of shared annotation terms. As an illustration, we have analyzed a gene set encompassing putative targets of the Engrailed transcription factor in *Drosophila melanogaster*. These genes were identified on the basis of *in vivo *UV cross-linking and chromatin immunoprecipitation experiments (X-ChIP) [11]. These experiments led to the cloning and sequencing of several hundreds of DNA fragments, allowing the computational identification of a well conserved DNA pattern, which was closely related to the known engrailed consensus. In order to delineate potential functional biases among engrailed targets, we have used Go-Diet and Go-Proxy to cluster the corresponding genes on the basis of 'Biological Processes' GO annotations.

In the first step, the set of putative target genes has been fed to the dataset-creation program and slimmed down by cutting the annotations to the fourth level of the Gene Ontology, using GO-Diet. This eliminates the poorly informative terms. In a second step, the resulting dataset has been processed with GO-Proxy, leading to 11 classes as shown in Table 2. Finally, for each of these classes, the probability of obtaining it by chance has been calculated, enabling the evaluation of the significance of the corresponding class relative to the initial gene dataset. In this analysis, the GOToolBox suite has proved to be very useful to define different functionally related sub-groups within a set of genes harbouring different functions (D.M., F. Maschat and B.J., unpublished work).

## Comparison of GOToolBox with other GO-based analysis programs

In this study, we have described the GOToolBox suite, which performs five main tasks: gene dataset creation, selection and fitting of ontology level (GO-Diet), statistical analysis of terms associated with gene sets (GO-Stats), GO-based gene clustering (GO-Proxy), and gene retrieval based on GO annotation similarity (GO-Family). Recently, several web-based GO-processing tools have been developed to display, query or process GO annotations. In this section, we are interested in comparing GOToolBox to several GO-processing programs. As shown in Table 3, comparisons were performed with 12 web-based programs listed on the official GO site [12].

### Functionalities unique to the GOToolBox suite

First, it should be highlighted that, to the best of our knowledge, no other program performing all five functions proposed in GOToolBox exists at present. Furthermore, the GO-Proxy and GO-Family tasks are unique to GOToolBox. These two functionalities are potentially very useful to the biologist. Indeed, on the one hand, the GO-Proxy implementation of a gene-to-gene distance calculation based on several GO terms allows the determination of classes consisting of functionally related genes. This feature should prove useful in all cases where the user wishes to identify functional subgroups within a list of genes of interest. On the other hand, the ability to search for genes similar to a user-defined gene on the basis of related GO terms (GO-Family) is also unique among all GO processing tools. When used to find functionally similar genes within a given species, the GO-Family program is often able to find paralogs as well as other genes with related functions, independently of sequence similarities. Similarly, when used to find functionally similar genes in other species, the program can successfully identify genes with related functions, including orthologs. In addition, the GO-Family program could be very valuable in the context of genome annotation: it could be used by database annotators to verify the coherence of the annotations of genes with known related functions, which if correctly annotated, would indeed be expected to be detected by the program.

Because of the presence of these two programs in our suite, we are inclined to think that GOToolBox represents a major improvement over other GO-based Web tools.

### Comparison of statistical analyses performed by all GO-based Web tools

Numerous programs have been developed to provide statistical evaluation of the occurrence of GO terms (Table 3). We compared these programs to GO-Stats at two levels: the statistics used to calculate the enrichment/depletion of GO terms, and the availability of different features, such as the output types and the GO terms filtering utilities to create the gene dataset.

As shown in Table 3 (column 3), four different approaches to calculating the probability of having *x *genes annotated for a given GO category have been implemented in various dedicated programs: hypergeometric distribution, binomial distribution, Fisher exact test and Chi-square test.

The two latter are non-parametric tests and are therefore less powerful than *P*-value calculations obtained with both the hypergeometric and the binomial distributions. In particular, the Chi-square test seems to be the less efficient, because it only gives valid results for large gene datasets, and it does not distinguish between over and under-represented terms [13].

The binomial distribution permits us to calculate the probability of obtaining *x *genes annotated for a given GO category when randomly picking *k *times one gene among *N *genes, leaving the possibility that one gene can be picked many times, which is not the correct situation in our case. It is important to note that when *N *is large, the hypergeometric distribution tends to give the same results as the binomial distribution. On average, the hypergeometric distribution seems to be both the most adapted model and the most powerful statistical test.

To compare the results obtained with the different methods for *P*-value calculation, we have implemented these methods in the GO-Stats module of GOToolBox, excepted the Chi-square test for reasons explained above. The implementation of these tests in GO-Stats permits us to compare the methods without having to deal with problems due to program-specific input formats, data update, and supported/unsupported organism species, as is often the case when using different programs. In addition, this gives great flexibility to the user, allowing he or she to use different statistical methods. We verified that (as might be expected) different programs using the same statistical methods give the same results. This was essentially true, with slight variations probably due to the use of different versions of GO by some programs (data not shown). Therefore, the comparison between programs mainly relies on the number of possible statistical tests that are available. As shown in Table 3, three programs (GOToolBox, GFINDer [13] and CLENCH [14]) propose the same three possible statistical tests, whereas all other programs have implemented only one method.

However, among these three programs, *GOToolBox *is the only one in which a multiple testing correction is implemented to adjust *P*-values and provide a correction for the occurrence of false positives. We choose the Bonferroni correction since it appears to be the most stringent in assessing the significance of enrichment/depletion

### Comparison of other features proposed by GO-based web tools

In addition to the statistical tests used by the different programs, the presence of functional features offering flexibility to the end-user can also be considered as a criterion for program comparison. Features such as the GO terms filtering utilities and output types proposed by different programs are worth comparing (Table 3, last two columns).

The GO terms filtering functions allows one to restrict the number of GO terms associated with each gene in the dataset, to facilitate interpretation of the results. Many ways to perform this restriction are possible: either mapping the terms on a slim ontology or fitting the terms to a given level (depth) of the ontology hierarchy. As shown in Table 3, only GOToolBox allows the use of both these filtering methods. They have been implemented and are accessible under the 'Create Dataset' form. In addition, in GOToolBox it is possible to restrict the number of terms associated with each gene, by taking into account only terms inferred in a particular way (for instance, terms inferred from direct assay) and to combine the filtering methods with the slim mapping or the level fitting described above.

As far as the output types are concerned, several programs propose a tabulated output file with terms ranked according to their *P*-values, (with the exception of GoMiner [15] and GOTM [16], therefore precluding the interpretation of the results in these cases). However, a positive attribute of GO Term Finder [17], GOTM and GoMiner over GOToolBox is that they propose directed acyclic graph (DAG) graphics for visualization of results. At the moment, GO-Stats allows the visualization of relationships between terms in tabulated output only, but a future version of GOToolBox will also incorporate a DAG graphical output option.

In conclusion, the GOToolBox is a multipurpose, flexible and evolvable software suite that compares favorably to all existing GO-based web-analysis programs. Its two unique features, GO-Proxy and GO-Family, enable new kinds of analyses to be carried out, based on the functional annotations of gene datasets These new functionalities are likely to be very useful to many biologists wanting to extract novel and meaningful biological information from gene datasets.

## Figures and Tables

**Table 1:** Examples of class relevance evaluation

Original class annotations	Most relevant term among class annotations	Associated probability	Number of proteins in the class	Number of class proteins annotated for the term	Number of proteins annotated for the term in the reference set
Conjugation with cellular fusion; perception of abiotic stimulus; cell surface receptor linked signal transduction; sensory perception; response to pheromone during conjugation with cellular fusion	Conjugation with cellular fusion	3.85E-09	11	8	32
RNA metabolism	RNA metabolism	2.76E-09	32	17	70
Cell cycle	Cell cycle	0.07383	6	3	115

The second and third columns are the results of the GO-Stats program, whereas all other columns are the results of a PRODISTIN analysis (see text for details).

**Table 2:** Classes found by *GO-Proxy *in a set of transcriptional putative targets and their statistical evaluation

Class-associated term	Number of genes in the class	Probability
Neurophysiological process	8	5.642e-9
Nucleobase, nucleoside, nucleotide and nucleic acid metabolism	7	2.609e-8
Cell growth and/or maintenance	14	1.011e-7
Protein metabolism	7	0.000001
Organismal movement	5	0.000006
Organogenesis	6	0.000030
Phosphorus metabolism	4	0.000110
Cell adhesion	3	0.000302
Response to external stimulus	3	0.001510
Signal transduction	5	0.002765
Signal transduction	3	0.034355

**Table 3:** Summary of the functionalities offered by GOToolBox and other GO processing tools

Program	References	Statistics	Multiple testing correction	Output	Ontology options
eGOn	[18]	Fisher exact test	-	TAB/RANK/TREE	ALL/EVID
CLENCH	[14]	Hypergeometric Binomial Chi Square	-	TAB/RANK	ALL/SLIM
FatiGO	[19]	Fisher exact test	Westfall/Benjamini/Yekutieli	TAB/RANK/TREE	LEVEL
FuncAssociate	[20]	Fisher exact test	*P*-value adjustment	TAB/RANK	ALL
FuncSpec	[21]	Hypergeometric	Bonferroni	TAB/RANK	ALL
GeneMerge	[22]	Hypergeometric	Bonferroni	TAB/RANK	ALL
GFINDer	[13]	Hypergeometric Fisher exact test Binomial	-	TAB/RANK	ALL
GoMiner	[15]	Fisher exact test	-	TAB/DAG/TREE	ALL
Gostat	[23]	Fisher exact test	Holm/Benjamini/Yekutieli	TAB/RANK	LEVEL
GO Term-Finder (CPAN)	[17]	Hypergeometric	Bonferroni/Benjamini	TAB/RANK/DAG	ALL
GO Term-Finder (SGD)	[17]	Binomial	-	TAB/RANK/DAG	ALL
GOTM	[16]	Hypergeometric	-	TAB/TREE/DAG	ALL
GOToolBox	This paper	Hypergeometric Fisher exact test Binomial	Bonferroni	TAB/RANK	ALL/SLIM/ LEVEL/EVID

In the output column, TREE, DAG, RANK and TAB refer respectively to tree-based output, directed acyclic graph visualization, *P*-value based ranking of terms, and results organized in a table. In the Ontology options column, terms listed refer to the way a gene set-GO term association can be built: ALL stands for 'all terms are taken into account (including parent terms)'; SLIM for 'mapping of the terms on a slim ontology'; LEVEL for 'fit the terms to a given depth of the ontology'; and EVID for 'filter terms according to the type of evidence which indicates how annotation has been associated to the gene'. See text for more details.
